# Therapeutic potential of bone marrow mesenchymal stem cells in cyclophosphamide-induced infertility

**DOI:** 10.3389/fphar.2023.1122175

**Published:** 2023-03-22

**Authors:** Dalia Ibrahim, Nadia Abozied, Samar Abdel Maboud, Ahmad Alzamami, Norah A. Alturki, Mariusz Jaremko, Maram Khalil Alanazi, Hayaa M. Alhuthali, Asmaa Seddek

**Affiliations:** ^1^ The Department of Physiology, Faculty of Medicine, Suez Canal University, Ismailia, Egypt; ^2^ The Department of Pharmacology, Faculty of Medicine, Suez Canal University, Ismailia, Egypt; ^3^ The Department of Pathology, Faculty of Medicine, Suez Canal University, Ismailia, Egypt; ^4^ Clinical Laboratory Science Department, College of Applied Medical Science, Shaqra University, AlQuwayiyah, Saudi Arabia; ^5^ Clinical Laboratory Science Department, College of Applied Medical Science, King Saud University, Riyadh, Saudi Arabia; ^6^ Smart-Health Initiative and Red Sea Research Center, Division of Biological and Environmental Sciences and Engineering, King Abdullah University of Science and Technology, Thuwal, Saudi Arabia; ^7^ Pharm.D, Scientific Office and Regulatory Affair Department, Dallah Pharma Company, Riyadh, Saudi Arabia; ^8^ Department of Clinical laboratory sciences, College of Applied Medical Sciences, Taif University, Taif, Saudi Arabia

**Keywords:** infertility, testicular injury, histopathology, MSCs, oxidative stress, sperm

## Abstract

Cancer is a deadly disease characterized by abnormal cell proliferation. Chemotherapy is one technique of cancer treatment. Cyclophosphamide (CYP) is the most powerful chemotherapy medication, yet it has serious adverse effects. It is an antimitotic medicine that regulates cell proliferation and primarily targets quickly dividing cells, and it has been related to varying levels of infertility in humans. In the current study, we assessed the biochemical, histological, and microscopic evaluations of testicular damage following cyclophosphamide administration. Further, we have explored the potential protective impact of mesenchymal stem cell (MSCs) transplantation. The biochemical results revealed that administration of cyclophosphamide increased serum concentrations of follicle-stimulating hormone (FSH) and luteinizing hormone (LH), while it decreased serum concentrations of free testosterone hormone (TH), testicular follicle-stimulating hormone, luteinizing hormone, and free testosterone hormone concentrations, testicular total antioxidant capacity (TAC), and testicular activity of superoxide dismutase (SOD) enzyme. The histology and sperm examinations revealed that cyclophosphamide induced destruction to the architectures of several tissues in the testes, which drastically reduced the Johnsen score as well as the spermatogenesis process. Surprisingly, transplantation of mesenchymal stem cell after cyclophosphamide administration altered the deterioration effect of cyclophosphamide injury on the testicular tissues, as demonstrated by biochemical and histological analysis. Our results indicated alleviation of serum and testicular sex hormones, as well as testicular oxidative stress markers (total antioxidant capacity and superoxide dismutase activity), and nearly restored the normal appearance of the testicular tissues, Johnsen score, and spermatogenesis process. In conclusion, our work emphasizes the protective pharmacological use of mesenchymal stem cell to mitigate the effects of cyclophosphamide on testicular tissues that impair the spermatogenesis process following chemotherapy. These findings indicate that transferring mesenchymal stem cell to chemotherapy patients could significantly improve spermatogenesis.

## 1 Introduction

Cancer is a deadly disease marked by abnormal growth of cells, altered gene expression, and unrestricted proliferation, leading to the production of malignant cells and initiation of metastasis or tissue invasion (Multhoff et al., 2012; Al-Abkal et al., 2022). Chemotherapeutic drugs, radiotherapy, immunotherapy, hormonal therapy, and surgical methods are all different therapies for treatment of cancer disease (Freireich et al., 1966; Alam et al., 2018; Gaber et al., 2020; Taniura et al., 2020; Basak et al., 2021; El Azab et al., 2021; Salem et al., 2022). The seminiferous epithelium is extremely vulnerable to toxicant damage, notably chemotherapy and radiation employed in cancer treatment. Consequently, young cancer survivors frequently experience persistent and possibly permanent declines in sperm counts, known as azoospermia, subsequently becoming infertile. Testicular tissues are susceptible to chemotherapeutic medicines as well as radiation as the developing spermatogonia are always mitotically active. The loss of these cells causes the development of the depletion of later stages of germ cells, as well as a decrease in sperm counts (Drumond et al., 2011).

Cyclophosphamide (CYP) is an effective chemotherapy, but it has detrimental side effects. It is an antimitotic drug that influences cell proliferation and specifically targets rapidly dividing cells (Ahlmann and Hempel, 2016; Bertholee et al., 2017; Basak et al., 2021). In the liver, enzymes such as cytochrome P450 metabolise CYP to its responsive mediates, phosphoramide mustard and acrolein, which induce DNA mutations (Ramadan et al., 2011; Hosseini et al., 2018; Fusco et al., 2021). CYP has mutagenic effects on the male genital system, causing the lack of spermatogenesis process in the testes, as well as damage to spermatogenic cells and decreased fertility (Adana et al., 2022). CYP’s detrimental effects include the obstruction of new Leydig cell development, as well as a decrease in sex hormone synthesis and infertility owing to spermatogonia depletion. Its poisonous influences on the testicular tissues were primarily assigned to oxidative damage on the Sertoli cells as well as seminiferous tubules, which impaired androgenesis and sperm production that caused programmed death for germ cells. Accordingly, using CYP in adult male rats caused a drop in the mass of the genital tissues, which may imply a decrease in the synthesis of these cells and a reduction in their reproductive ability (Al-Niwehee and Al-Rudaini, 2019). Agreeing with Cao et al. (2017), CYP can affect tissue redox balance, implying that oxidative stress might cause biochemical and physiological problems. In our current study, we selected CYP as it is widely employed in chemotherapy and has been linked to varying incidences of infertility in people.

Stem cell transplantation has emerged as a novel curative approach in repairing organ or tissue architecture and functions. Mesenchymal stem cells (MSCs) are cells able to be renewable and can differentiate into many lineages. MSCs could be separated from a wide range of tissues, including bone marrow, endometrial colonies, menstrual blood, adipose tissue, and umbilical cords, and are easy to separate, proliferate rapidly, and can differentiate into other cells (Ding et al., 2011; Cakici et al., 2013; Zhang et al., 2014). MSC-secreted growth factors have been demonstrated to play a role in cell endurance, proliferation, migration, angiogenesis, and immunological regulation; as a result, these cells have been proposed as qualified applicants for regenerative medicine. These cells might enter injured tissue *via* the circulation and differentiate into cells that are identical to target tissue cells (Drumond et al., 2011; Fazeli et al., 2018; Rühle et al., 2019). MSCs account for a very small percentage of the nucleated cells in bone marrow, and although MSCs are uncommon, they may be clearly distinguished from hematopoietic stem cells (HSCs) as they can be grown *ex vivo*. The MSCs’ multi-potency has now been firmly proven, and several differentiated cell lines have been produced from them. Additionally, this cell type may be important in endogenous tissue healing, and MSCs have recently been considered as a viable cellular environment for tissue production and cellular treatment for tissue regeneration and reconstruction (Gnecchi et al., 2009; Gomez-Salazar et al., 2020).

MSCs exhibit a variety of germ cell (GC) features and can be encouraged to develop into GCs in the presence of specific stimuli. Because of these distinguishing impacts, GCs were considered as a potential candidate for the cellular treatment MSCs. As a result, infertility investigators are driven to employ these kinds of cells to be a prospective treatment for infertility. To generate GCs from MSCs, various stimulators were used, and these cells were transplanted into gonads as they have obvious roles in cell growth, differentiation, GC generation, and GC meiosis and gametogenesis (Ghasemzadeh et al., 2015).

Infertility is caused by cancer treatment methods utilised during infancy in a large number of male children cancer patients. Spermatogonial stem cells (SSC) are the sole option for preserving fertility because spermatogenesis does not begin in infancy. Sertoli cells promote SSC self-renewal and differentiation. The male undifferentiated GC pool is made up of SSCs (Singh et al., 2011). Mesenchymal stem and Sertoli cells generated from bone marrow share a common embryonic derivation, differentiation, and immunomodulatory potential. Their proliferation and gene expression profile are crucial in spermatogenic control (Önen et al., 2022). SSCs are kinds of cells located in the testes which exist near the basement layer of seminiferous tubules. They can divide through mitosis to produce an ever-massive influx of developing germ cells. SSCs provide billions of sperms every day during a male’s reproductive life due to their critical function in sustaining spermatogenesis. SSCs generate daughter germ cells that go through coordinated, progressive, and intensive differentiation procedures to be changed from spherical cells into sperms. The spermatogenesis process is a complicated one of proliferation of GC and differentiation that involves significant relations between numerous cell types, hormones, and growth factors (Aliakbari et al., 2016; Chen et al., 2020; Ibtisham and Honaramooz, 2020). Stem cells could have helped to restore spermatogenesis in two ways: sustaining pre-existing SSCs or transdifferentiating into SSC-like cells to create spermatocytes (Cakici et al., 2013). Stem cells are relocated to injured testicular regions, decreasing the amount of apoptotic Leydig cells and enhancing serum testosterone. Elevated ROS and impaired antioxidant defence cause redox instability and decreased mobility of sperms, and cause injury to sperm DNA. Sperms are particularly vulnerable to the damaging effects of ROS because of the high concentrations of unsaturated fatty acids that are found in the cellular membrane (Alahmar, 2019). ROS increases lipid peroxidation, leading to intracellular oxidative stress. The process involves lipid peroxidation, membrane stability loss with enhanced permeability, decreased mobility of sperms, DNA injury, and ultimately apoptosis (Fusco et al., 2021; Abdel-Wahab et al., 2022; Khirallah et al., 2022a; Khirallah et al., 2022b). A number of inherent and exogenous variables have been linked to improved OS in the male reproductive system. The initial line of defence against oxygen-free radicals is superoxide dismutases (SODs). Changes in SOD expression or activity have been identified in a variety of clinical situations (Oyagbemi et al., 2016). The function of MSCs in reducing oxidative damage has attracted a lot of interest. MSC treatment has been shown to have antioxidant properties in a variety of disease types as MSCs can immediately alleviate oxidative stress-associated damage. Because oxidative stress is linked to cell damage, inflammation, and metabolic dysfunction, it is a crucial pathophysiological process in many disorders (Emwas et al., 2019; Chandra et al., 2021; Lutz and Bernard, 2022). Oxidative stress, along with redox instability, is caused by chemical elements found in all living cells that perform comparable activities. Thus, MSCs’ capacity to control these processes may explain the range of disease models curable by MSCs (Stavely and Nurgali, 2020).

Supported by the data above, we set out to investigate the biochemical and microscopic bases of cyclophosphamide-induced infertility by toxicities in testes. In addition, by using biochemical, histological, and microscopic inspection approaches, we studied MSCs’ potential therapeutic capacity versus cyclophosphamide-induced infertility by toxicities in testes.

## 2 Materials and methods

### 2.1 Animals

Our research is an investigational case-control study, the Institutional of Animal Ethics Committee in Suez Canal University’s Faculty of Medicine, Physiology department, authorised the animal research (protocol code 4,709). 30 Sprague-Dawley mature male albino rats, their age was 3 months old (180–200 g) kept in animal sanctuary in faculty of medicine Suez Canal university and rats were acclimatised for 7 days under room temperature-controlled circumstances (20°C ± 2°C) with unrestricted access to normal pellet animal feed and tap water and 12 h daylight cycle control.

### 2.2 Chemicals and reagents

Endoxan 1 g vial (Cyclophosphamide, 1,000 mg, Baxter Co., Deerfield, Illinois, United States). In addition, phosphate buffer saline (PBS), trypan blue, 70% ethyl alcohol, Dulbecco’s Modified Eagle Medium (DMEM), diethyl ether, all were acquired *via* Sigma Aldrich Co. Ltd (St. Louis is in Missouri, United States).

### 2.3 Experimental strategy and groups

#### 2.3.1 Induction of male infertility

To induce infertility in male rats, cyclophosphamide (CYP) was administrated intraperitoneally (i.p) with one dose (50 mg/kg bw) for a day then followed by daily dose (8 mg/kg bw) about 14 days using ampoule of Endoxan 1 g (Baxter Co.), that dispersed in phosphate buffer saline (PBS) (Abbasy et al., 2010).

#### 2.3.2 Transplantation of mesenchymal stem cells

The remedy of male infertility was performed by injection in both rats’ testes with 0.2 mL of PBS combined to the stem cell pellet (1 × 106 cells/rat) following the final cyclophosphamide dose (Gundersen and Jensen, 1987; Dissassa et al., 2013).

#### 2.3.3 Animals grouping

The animals were assigned at random to three equivalent groups (n = 6):

Group I: Control group.

Group II: (CYP); Cyclophosphamide-treated group, animals were subjected to induction of male infertility *via* cyclophosphamide (individual dosage of 50 mg/kg bw/1 day afterward daily dose of 8 mg/kg bw/2 weeks, i.p.).

Group III: (CYP + MSCs); Cyclophosphamide-treated group and then transplanted with mesenchymal stem cells (1 × 10^6^ cells/rat/3 weeks) in the rat both testes, after the last injection of cyclophosphamide (Abbasy et al., 2010).

### 2.4 Isolation of stem cells

#### 2.4.1 Separation of bone marrow stem cells (BMSCs)

Hind limbs from rats were collected and cleaned from skin, muscles, and connective tissue. The limbs above the pelvis and below the ankle bone were severed; it is critical to preserve the bone ends to assure the purity of the bone marrow. After cutting the limbs, the knee joint was gently separated. The bones were then washed with gloved fingers soaked in 70% ethyl alcohol and put in a plate of sterile PBS on ice before being transported into a sterile tissue culture hood (BIOPTIMA series-biological safety cabinet–Telstar catalog no. EQM-SCBI, Bosschendijk 215. 4731 DD Oudenbosch, Netherlands). Bones were washed by moving through sterile PBS six intervals. Ends of each bone were sniped off with scissors and gently place in sterile PBS. With forceps, grab one bone. Media was forced by syringe through bone shaft to remove all red marrow into 150 mm plate. This procedure was performed several times to guarantee that all marrow was extracted. Continue until all bones are de-marrowed. Cell mixture was pipetted several times to separate cells; a syringe was used to extract huge marrow fragments through the needle to further separate them. Centrifugation was done at 1,500 rpm through 5 min and the supernatant was taken then taken. The cell pellet was resuspended with 5.0 mL PBS and was combined using a Pasteur pipet and gentle aspiration. Centrifugation was done at 1,500 rpm for 5 minutes. Finally, the amount of the split cells was calculated by Countess™ three Automated Cell Counter (Thermo Fisher Scientific Inc (Waltham, Massachusetts, United States), Catalog no. AMQAX 2000) and the viability was established by the trypan blue elimination test (Gnecchi et al., 2009).

#### 2.4.2 Culture for mesenchymal stem cells (MSCs)

Cells were cultured in flasks of 25 cm^2^ at a concentration of (1 × 10^6^) mononuclear cells/cm^2^, and then were cultivated with DMEM added with 1% glutamine, 1% antibiotic/antimycotic and 20% fetal bovine serum. After that, cells were kept at 37°C, humid environment including 5% CO_2_. Non-adherent cells were eliminated after a day of incubation, then clean DMEM media was applied to the culture flask. Subsequently, the culture media was substituted every 4 days, and cellular development was monitored daily using an inverted microscope. The cells were extracted when they achieved 50%–60% proliferation after being treated with 0.025 percent trypsin/ethylene-diamine-tetra-acetic acid (EDTA).

### 2.5 Biochemical analysis

After 3 weeks of the transplantation of stem cells, semen analysis done for all rats and then they were anesthetized with diethyl ether. From the caudal vein and retro-orbital venous plexus, we took the blood using capillary tubes from all rats, centrifuged to obtain serum. Serum was removed and kept at −80°C for further examinations of serum follicle-stimulating hormone (FSH), serum luteinizing hormone (LH) and serum free testosterone hormone. Moreover, the testes were removed for histological examination and Jonson score and measurement of LH, FSH and testosterone hormone and evaluation of total antioxidant capacity (TAC) and the activity of superoxide dismutase (SOD) enzyme in testicular tissue. For tissue homogenate, testes samples were homogenized thoroughly with PBS, then centrifuged at 2,000–3,000 rpm/20 min to gather supernatant that was kept at −80°C.

#### 2.5.1 Assessment of serum hormones

Serum was kept for analysis of FSH, LH, and free testosterone *via* Enzyme-Linked Immunosorbent Assay (ELISA) kits. FSH was estimated by Rat Follicle-Stimulating Hormone (FSH) ELISA Kit, LH was estimated by Rat luteinizing hormone (LH) ELISA kit, free testosterone was assessed utilising rat free testosterone ELISA kit. All the kits were purchased from My BioSource, Inc (San Diego, CA, United States) with catalog no. MBS2502190, MBS764675 and MBS8807666, respectively.

#### 2.5.2 Assessment of testicular hormones

Tissues homogenates were kept for analysis for FSH, LH, and free testosterone by ELISA using the previous kits used for serum analysis.

#### 2.5.3 Assessment of testicular oxidative stress markers

Rat testicles were gathered then frozen instantly in liquid nitrogen then homogenized thoroughly with PBS, then centrifugation at the speed of 2,000–3,000 rpm for 20 min was done to gather supernatant, which was maintained at −80°C for analysis of testicular total antioxidant capacity (TAC) as well as activity of superoxide dismutase (SOD) enzyme using kit obtained from My BioSource, Inc (San Diego, CA, United States) with Catalog no. MBS2540515 and MBS822352, respectively.

### 2.6 Histological assessment

Testes were obtained, divided into blocks, and preserved in a formalin buffer solution containing 10% formalin. Following preservation, the blocks were immersed in paraffin, and histological slices (5 m thick) were sliced from each block and dyed with hematoxylin-eosin (Mohamed et al., 2021). Sections were shot and viewed using a light microscope (Model CX21; Olympus, Tokyo, Japan) and an adapted digital camera (AM423U Eyepiece Camera; Dino-Eye, San-Chung, Taiwan).

Johnsen score: Slides were inspected with a light microscopy (magnification power, ×100) to determine the Johnsen score. Each tubule counted received a score. The score was multiplied by the quantity of tubules with a specific score. The sum of the individual assessments was therefore divided by the number of tubules examined to calculate the Johnsen score (Supplementary Table S1). A score of ten implied maximal spermatogenesis process, whereas a score of one shows total lack of germ cells (Torabi et al., 2017; Teixeira et al., 2019; Mohamed et al., 2021; Mohamed et al., 2022a; Mohamed et al., 2022b).

### 2.7 Semen analysis

In sterile containers, semen samples were assembled and let to solubilize at 37°C/half an hour (World Health Organization, 2010; Teixeira et al., 2019). Sperm count, viability, as well as motility were examined using a microscope equipped with a ×20 positive phase contrasting lens and a amplification of ×200. The number of spermatozoa per area corresponding to 1 mL was counted manually on every slide, and the data was represented as the estimated sperm concentration (×10^6^ spermatozoa/mL of semen). The motility of the sperms was estimated by observing 200 spermatozoa from at least five microscopic fields, our observation was to measure the total motility (any sort of movement: Rapid or slow progressive motility, as well as the non-progressive motility) of the sperms, in addition to their progressive motility (either rapid or slow progressive motility). To differentiate between live and dead cells, LIVE/DEAD™ Sperm Viability Kit (Catalog number: L7011, Thermo Fisher Scientific) was used to stain the sperms. The kit comprises two different dyes, SYBR 14 (1 mM) stained all cells because it is membrane permeable, but propidium iodide (PI) (2.4 mM) selectively stained dead cells because it only enters membrane-compromised cells (Holman, 2009).

### 2.8 Statistical analyses

First, size of samples was identified using G*Power software version 3.1.9.7 (Jacob, 1988; Faul et al., 2007). (n = 6). All assessments were recorded as mean ± S.E.M. Data was gathered, verified, reviewed, and organized in tables and figures employing the GraphPad prism software program (version 7.0 (2016) Inc., San Diego, CA, United States). Results data were evaluated for normality utilizing the Shapiro–Wilk normality test. Statistical significance in all groups was described by employing one-way ANOVA, next Duncan’s multiple range (DMRTs) *post hoc* test. Additionally, the discrete data were analysed applying the Kruskal–Wallis test (Hammer et al., 2001; Kalinichenko et al., 2021).

## 3 Results

### 3.1 Biochemical assessment

#### 3.1.1 Assessment of serum hormones

**FIGURE 1:**
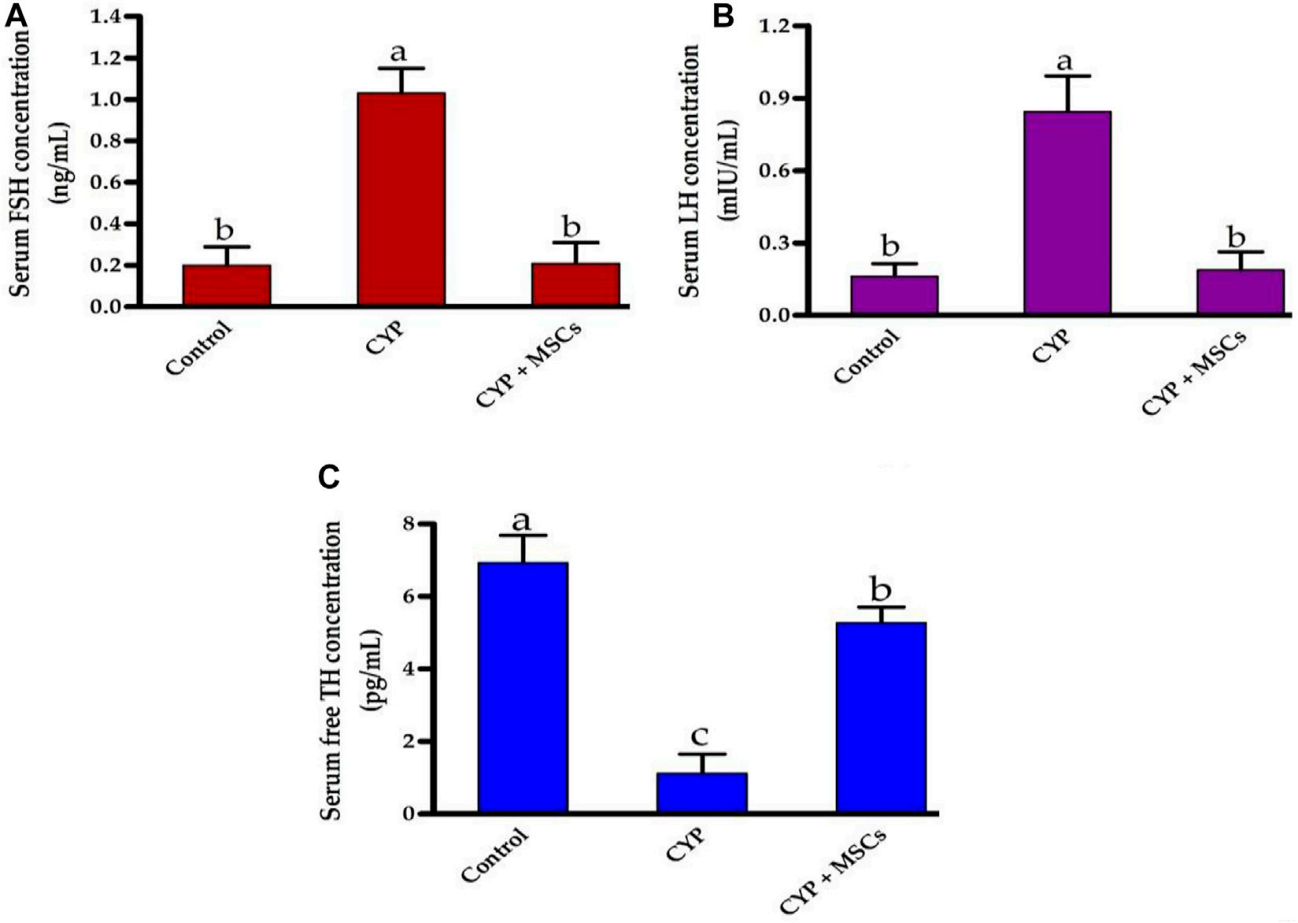
Influences of CYP with/without MSCs transplantation on serum hormones concentrations; **(A)** serum FSH, **(B)** serum LH, and **(C)** serum free TH. Data described as mean ± SD (n = 6). DMRTs show that bars with various letters are substantially different at *p* = 0.05.

Initially, we explored the effect of CYP treatment on the efficacy of testes by estimating the serum FSH (ng/mL), LH (mIU/mL), and free TH concentrations (pg/mL). As shown in Figures 1A, B, CYP therapy caused significant enhancement at *p* = 0.05 in the concentrations of FSH (ng/mL) and LH (mIU/mL) compared with the control group. Our current findings may relate to the damage that occurred in the testes by CYP that led to a decrease in testicular hormones and hence, a pituitary gland increase in the production of these hormones (FSH and LH). In contrast, Figure 1C showed that CYP treatment produced a significant decline at *p* = 0.05 in the concentrations of free TH (pg/mL) compared with the control group. Our current results revealed the cytotoxic impact of CYP on testicular tissues that caused a decrease in the production of the testosterone hormone. Subsequently, we studied the influence of the transplantation of MSCs after CYP treatment on the concentrations of serum FSH, LH, and free TH. Interestingly, transplantation of MSCs in testes tissues caused a significant reduction at *p* = 0.05 in the level of serum FSH (ng/mL) and LH (mIU/mL) compared with the CYP-treated group. Our current results indicated that MSCs caused restoration to the testicular damaged tissue. In contrast, transplantation of MSCs in testes tissues caused a significant rise at *p* = 0.05 in the level of serum free TH (pg/mL) compared with the CYP-treated group. Our current results showed the modulatory effect of MSCs on the testicular tissues that allowed the testes to produce testosterone hormone.

#### 3.1.2 Assessment of testicular hormones

**FIGURE 2:**
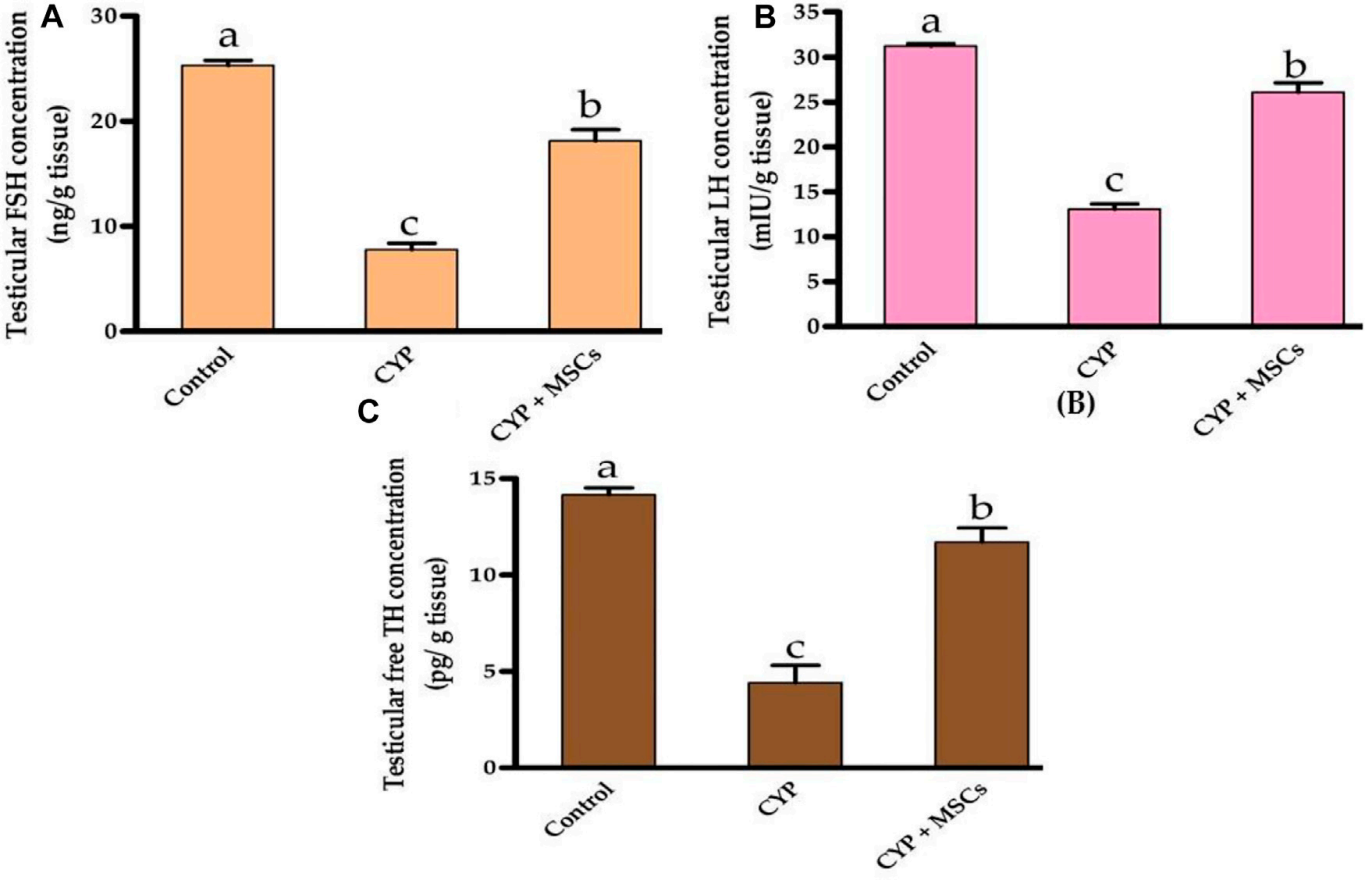
Influences of CYP with/without MSCs transplantation on testicular hormones concentrations; **(A)** testicular FSH, **(B)** testicular LH, and **(C)** testicular free TH. Data described as mean ± SD (n = 6). DMRTs show that bars with various letters are substantially different at *p* = 0.05.

Regarding the assessment of the CYP treatment effect on testicular tissue hormones, we evaluated the concentration of testicular FSH (ng/g tissue), LH (mIU/g tissue), and free TH (pg/g tissue). As demonstrated in Figures 2A–C, the concentrations of the testicular FSH (ng/g tissue), LH (mIU/g tissue), and free TH (pg/g tissue) were significantly reduced at *p* = 0.05 in the group treated with CYP compared with the control group. Our current results may affirm the infertility effect of CYP treatment that affects the hormones in the testicular tissues. The transplantation of MSCs in testicular tissues after CYP treatment triggered a significant rise at *p* = 0.05 in the concentration of the testicular FSH, LH, and free TH compared with the CYP-treated group. These findings might be attributed to MSCs restoring hormone synthesis by regulating the deleterious effect of CYP therapy.

#### 3.1.3 Assessment of testicular oxidative stress markers

**FIGURE 3:**
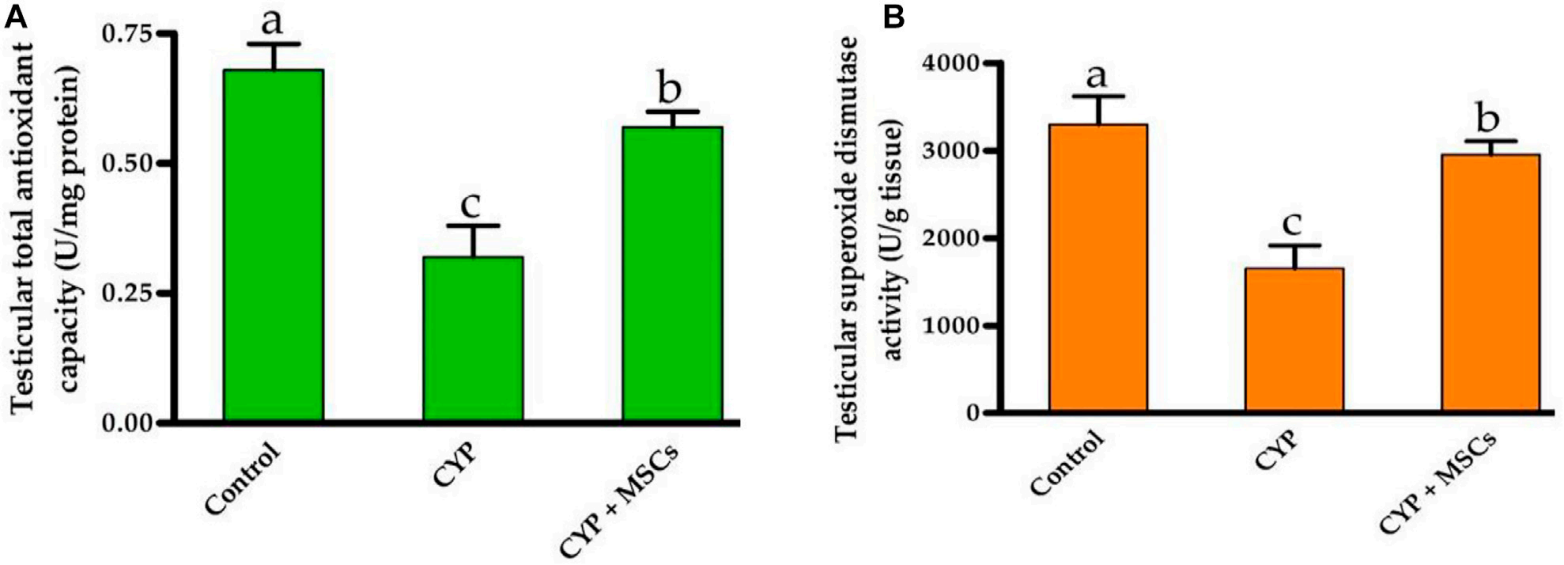
Influences of CYP with/without MSCs transplantation on testicular oxidative stress markers; **(A)** testicular total antioxidant capacity, **(B)** testicular superoxide dismutase activity. Data described as mean ± SD (n = 6). DMRTs show that bars with various letters are substantially different at *p* = 0.05.

To evaluate the influence of CYP with/without MSCs on oxidative stress markers in testicular tissue, we evaluated the total antioxidant capacity (U/mg protein) and the activity of the SOD enzyme (U/g tissue) in testicular tissues. As demonstrated in Figures 3A, B, the group treated with CYP demonstrated a significant reduction *p* = 0.05 in testicular total antioxidant capacity (U/mg protein) and in the activity of the SOD enzyme (U/g tissue) compared with the control group. Our current results affirmed the damage caused by CYP in the establishment of free radicals that cause oxidative stress in the tissues and formation of ROS. We then examined the ameliorative effect of MSCs after CYP on the oxidative stress markers in testicular tissue. We discovered that the CYP group treated with MSCs indicated a significant rise at *p* = 0.05 in testicular total antioxidant capacity (U/mg protein) and in the activity of the SOD enzyme (U/g tissue) compared with the CYP-treated only group. These findings revealed that MSCs have a protective effect on the tissues for the oxidative stress caused by the CYP treatment.

### 3.2 Histological examination

To confirm our biochemical findings, we used light microscopic analysis of histological alterations in testes sections stained by H&E to establish the effect of CYP therapy on testicular tissues, as well as the probable protective effect of MSC transplantation. We recorded our data according to Johnsen scores in the different groups.

**FIGURE 4:**
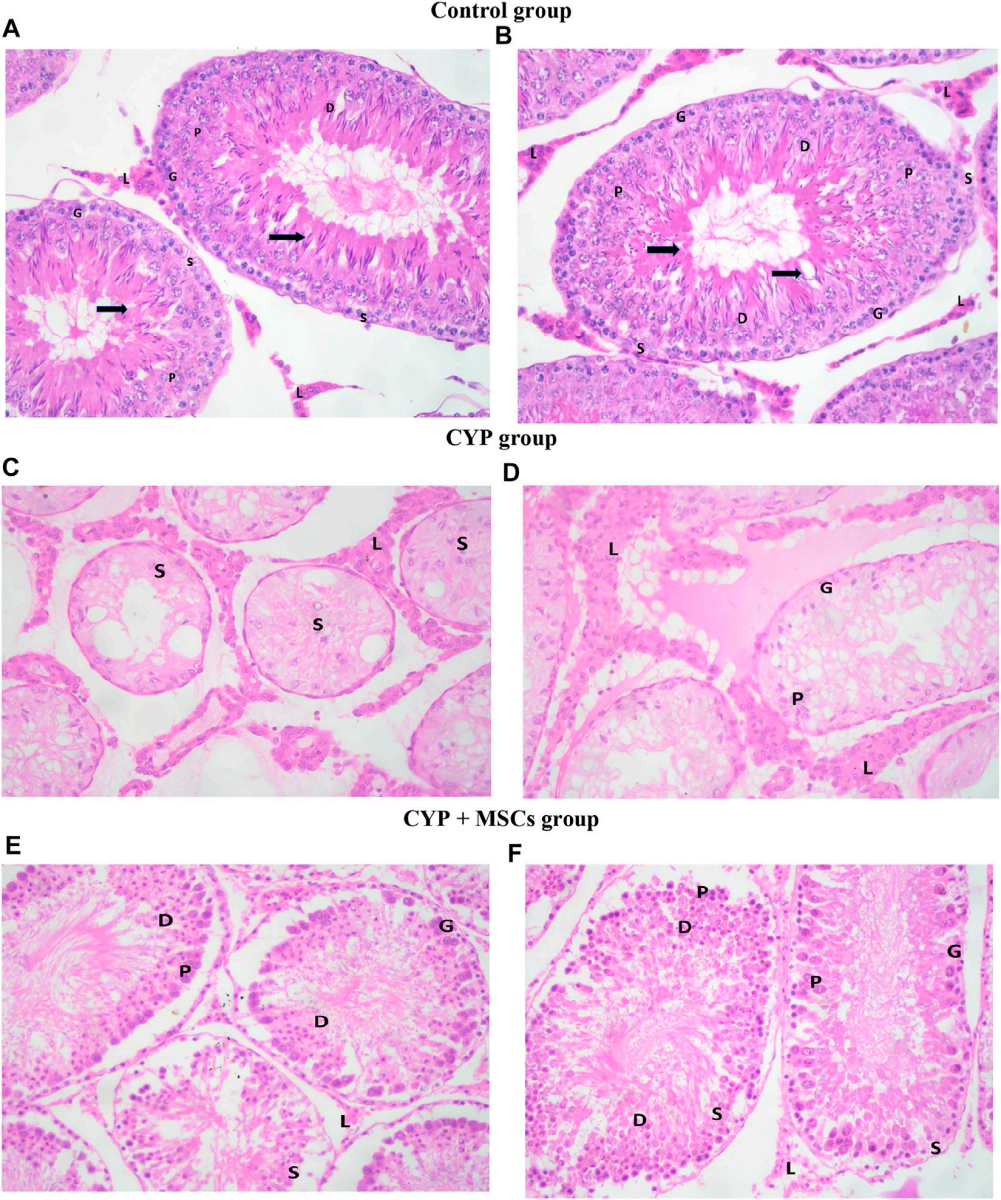
Photomicrograph of H&E-stained testis sections **(A, B)** control group showing seminiferous tubules. Spermatogonia cells (G) and Sertoli cells (S) lie on a normal basement membrane that is bordered by myoid cells (''). Primary spermatocytes (P), early spermatids (D), and spermatozoa (black-thick arrow) may be detected inside the lumen. Between the tubules, there are groups of interstitial Leydig cells (L) with vesicular nuclei **(C, D)** CYP group demonstrating lobules of seminiferous tubules showing almost only Sertoli cells (S), very few spermatogenic cells (G) and primary spermatocytes (P) with no evidence of spermatids and no evidence of maturation in most of the seminiferous tubules. There are many individual and groups of Leydig cells in the interstitial stroma (L) **(E, F)** CYP + MSCs group showing lobules of seminiferous tubules with spermatogenic cells (G) and Sertoli cells (S), primary spermatocytes (P) and few scattered early spermatids (D) with no evidence of maturation in most of the seminiferous tubules. There are few scattered individual and groups of Leydig cells (L) in the interstitial stroma. H&E-stained sections ×400.

H&E-stained control testes sections displayed firmly filled seminiferous tubules split by small interstitial gaps containing Leydig interstitial cell clusters with vesicular nuclei A distinct basement membrane, and flattened myoid cells with flattened nuclei surrounding each seminiferous tubule. Each tubule was bordered by spermatogonia, primary spermatocytes, early and late spermatids, and stratified germinal epithelium, and showed evidence of spermatogenesis with primary and secondary spermatocytes, spermatids, late spermatids, and scattered mature sperms in most of the seminiferous tubules. Sertoli cells were found among the spermatogenic cells, sitting on a normal basement membrane. They were characterised by large, faint vesicular nuclei. Moreover, spermatozoa were similarly discovered in the lumen of seminiferous tubules (Figures 4A, B. In the CYP group, H&E-stained sections showed different histological abnormalities in the seminiferous tubules than in the CYP treated group, such as decreases in the quantities of germ cells at different phases of spermatogenesis, with a low abundance of sperms detected in the seminiferous tubules. Deterioration of epithelial cells in seminiferous tubules was noted, with degradation of Sertoli cells, enlargement of the interstitial area, and vacuolization in interstitial tissues, as well as atrophy in the majority of Leydig cell tubules (Figures 4C, D. In the CYP + MSCs group, H&E-stained testes sections indicated less destruction than the CYP-only treated group, indicating that MSC transplantation in testes tissues alleviated the CYP-induced pathological lesions to some significant degree, with the appearance of thickened tunica albuginea, lobules of seminiferous tubules showing evidence of spermatogenesis with few scattered primary and secondary spermatocytes and spermatids, and many early spermatids in most of the seminiferous tubules with very few showing evidence of maturation along with scattered individual and sets of Leydig cells in the interstitial stroma (Figures 4E, F).

**FIGURE 5:**
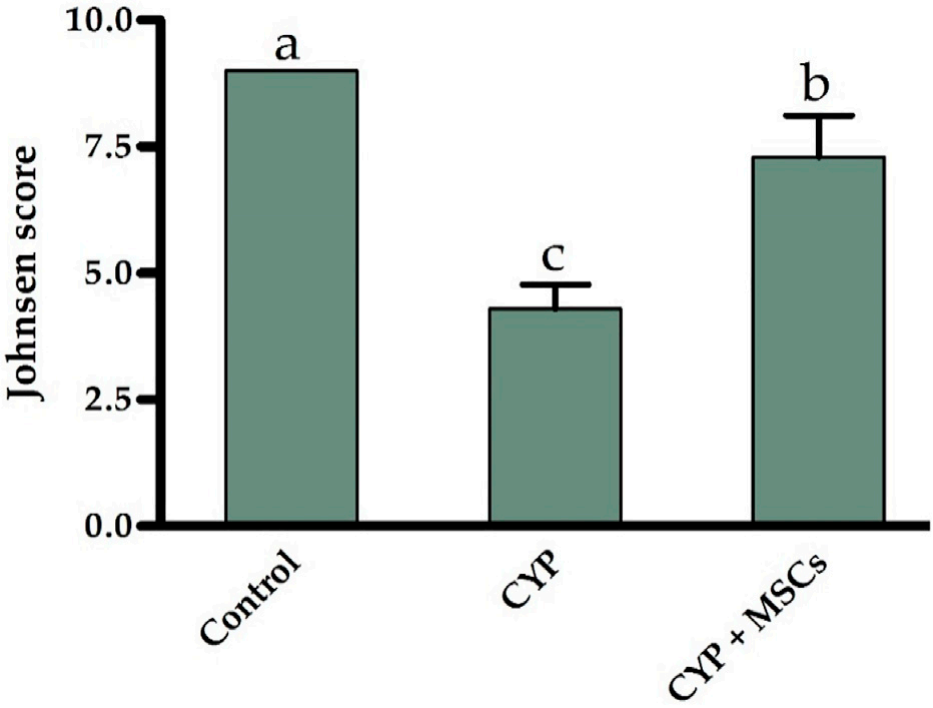
Influences of CYP with/without MSCs transplantation on Johnsen score for the seminiferous tubules. Data described as mean ± SD (n = 6). DMRTs show that bars with various letters are substantially different at *p* = 0.05.

Furthermore, based on the cell profile discovered along the seminiferous tubules, we utilized the Johnsen score to evaluate the spermatogenesis process. Figure 5 demonstrates that the spermatogenesis process according to the Johnsen score in the CYP-treated group was considerably diminished at *p* = 0.05 compared with the control group, and conversely, the spermatogenesis process according to the Johnsen score in the CYP group treated with MSCs was considerably developed at *p* = 0.05 compared to the CYP-treated only group. From the microscopic histological examination to the stained sections of testes and the estimation of the Johnsen score, we deduce that CYP treatment may cause infertility in male rats, and the transplantation of MSCs to CYP-treated rats improved the spermatogenesis process and protected the testes from CYP damage.

### 3.3 Semen analysis

**FIGURE 6:**
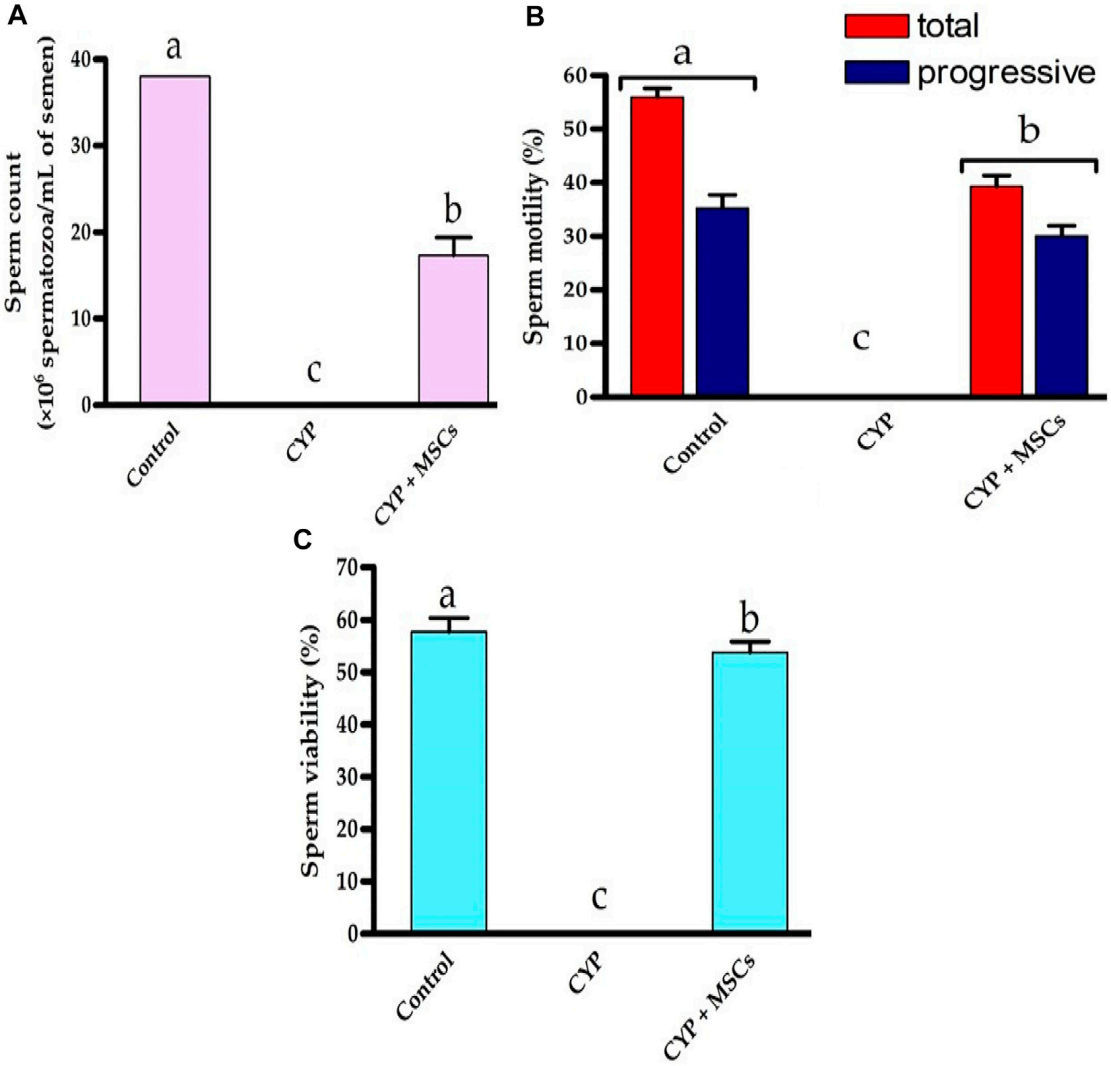
Influences of CYP with/without MSCs transplantation on semen analysis; **(A)** Sperm count (×10^6^ spermatozoa/mL semen), **(B)** sperm motility (%), **(C)** sperm viability (%). Data described as mean ± SD (n = 6). DMRTs show that bars with various letters are substantially different at *p* = 0.05.

For deep investigations for the effect of CYP treatment with/without MSCs, we examined the sperm count, percentage of mobility, and viability microscopically. Figures 6A–C demonstrated that the CYP treatment destroyed the sperms, as no sperms were discovered in the CYP-treated group’s semen study. On the other hand, transplantation of MSCs following CYP treatment guarded the sperms from the toxic influences of CYP therapy, although the sperm count (×10^6^ spermatozoa/mL of semen) was substantially lower at *p* = 0.05 compared with the control group. We discovered that the total and progressive mobility percentages of the sperms, as well as their viability percentage, were substantially lower in the CYP + MSCs treated group at *p* = 0.05 compared with the control group. These findings demonstrated that MSCs reduced the negative effects of CYP on spermatogenesis process.

## 4 Discussion

Throughout the life of a male, the spermatogenesis process sustains the generation of spermatozoa, the last cell carrier of genetic substances (Mruk and Cheng, 2004; Smith and Walker, 2014; Diao et al., 2022). Testicular damage is a significant issue following the treatment of different cancers, and infertility triggered by chemotherapy is a problematic issue that patients must deal with, and it may also have a negative influence on their family life (Cao et al., 2017). Chemotherapies have the propensity to produce harmful ROS, which can then affect adjacent tissue. ROS has several effects, including the inhibition of several enzymes, injury of DNA, and peroxidation of lipid, all of which promote infertility (Alahmar, 2019; Al-Abkal et al., 2022). ROS-induced oxidative stress (OS) is a familiar contributor to the aetiology of pathologic male infertility; indeed, augmented testicular oxidative stress can produce alteration in the forms of testicular micro-vascular blood flow and hormonal communication, causing an enhancement in germ cell death and, eventually, a decrease in the spermatogenesis process, believed to play a significant role in the pathophysiology of male infertility (Fusco et al., 2021; Al-Abkal et al., 2022). To retain reproductive capacity, some male cancer patients request sperm freezing operations prior to initiating treatment (Özatik et al., 2021). These individuals are in a terrible and stressful circumstance. This phenomenon, which has been reported as an adverse impact of CYP, is also exhibited in experimental animals (Özatik et al., 2021). In our current research, we explored the biochemical and histological results of testicular damage in CYP-treated rats by studying the prospective role of testicular tissue damage markers and attempted to discover the possible strategy of MSCs in restoring the tissue damage caused by CYP. To achieve these objectives, rats were injected with CYP (i.p) with a single dose (50 mg/kg bw) for a day then followed by a daily dose (8 mg/kg bw) for 2 weeks to elicit infertility.

CYP is converted by cytochrome P450 initially into two unconstant precursors, 4-hydroxycyclophosphamide and aldophosphamide, and then into two constant poisonous intermediates, phosphoramide mustard and acrolein (Ramadan et al., 2011). Phosphoramide mustard inhibits the division of cells by producing irreversible cross-linkages in DNA at guanine N-7 locations in DNA strands. Acrolein, conversely, is a reactive aldehyde with the capability of producing harmful ROS and thus affects adjacent tissues (Ganesan and Keating, 2015; Iqubal et al., 2019). CYP is commonly employed as an anti-cancer agent, but it can have a variety of side effects, such as genotoxicity, because it can impede cell growth due to its DNA-damaging effect (Zhu et al., 2015).

The anterior pituitary gland produces and releases FSH and LH, which attach to receptors in the gonads (ovaries or testes) and control testicular features by boosting the formation of sex steroids and gametogenesis. LH promotes testosterone hormone manufacture from the testicular interstitial cells in males (Leydig cells), while FSH promotes testicular growth and increases the manufacture of an androgen-binding protein by Sertoli cells, constituent with the testicular tubule required for sperm cell maturation (Babu et al., 2004; Oduwole et al., 2018). In our current research, CYP was injected intraperitoneally to stimulate infertility. It was noticed that CYP administration triggered a substantial rise in serum sex hormones (FSH, LH, and free TH), while triggering a substantial decline in serum-free TH as well as testicular sex hormones (TSH, LH, and free TH). These results indicated that CYP has a toxic effect on the anterior pituitary gland, essential for hormone production, causing a disturbance in hormone release (Oyagbemi et al., 2016). A decrease in serum testosterone could be caused by CYP-induced membrane lipid peroxidation in the testes, which is caused by an elevation in oxidative stress, producing injury to DNA, proteins, and enzymes implicated in testicular steroidogenesis and spermatogenesis. It is normal for testicular FSH, LH, and free TH levels to decrease following testicular dysfunction caused by CYP as testicular damage leads to damage of FSH and LH receptors in testes (Oyagbemi et al., 2016; Özatik et al., 2021).

ROS generation surpasses the antioxidant defense system’s scavenging capability, and results in redox imbalance, oxidative stress, lipid peroxidation, protein oxidation, reduction of sperm motility, and sperm DNA damage; moreover, tissue injury induced by ROS production and oxidative stress causes alterations in cellular function, and oxidative stress and the generation of ROS have been involved in the aetiology of CYP toxicity (Cigni et al., 2008; Arnaud et al., 2017; Fusco et al., 2021). Our results showed that CYP treatment in adult male rats caused a decrease in testicular total antioxidant capacity as well as in the activity of the testicular SOD enzyme compared with the control group. CYP metabolites are hypothesised to generate oxidative stress and direct endothelial capillary injury, resulting in protein, erythrocyte, and hazardous metabolite extravasation. In the incidence of these poisonous metabolites, endothelial cell disintegration caused direct impairment to capillary blood vessels, which may explain the drop in testicular total antioxidant capacity and SOD enzyme activity, Previous research has shown that acrolein interacts with the tissue antioxidant defence system and increases reactive oxygen free radicals and lipid peroxidation *via* inactivating microsomal enzymes (Dhesi et al., 2013; Ayshath et al., 2016; Hosseini et al., 2018; Ayza et al., 2020). For further investigation, we examined histological analysis for testes in the CYP-treated group and discovered that CYP-induced testicular impotence in the form of azoospermia and seminiferous tubule atrophy caused tissue injury due to its cytotoxicity by generating ROS that affected the architecture of the testes tissue and caused damage to Sertoli cells, Leydig cells, and spermatozoa, resulting in no sperms, resulting in infertility (Namasivayam et al., 2006; Comish et al., 2014; Zhu et al., 2015; Hosseini et al., 2018; Merwid-Ląd et al., 2021). As we discovered, CYP induces testicular injury, which is verified by the Jonson score. Microscopic examination of the CYP group revealed testicular tissue with unremarkable tunica albuginea, lobules of seminiferous tubules showing Sertoli cells, a few primary and secondary spermatocytes, a few with no evidence of spermatids, and no evidence of maturation in most of the seminiferous tubules. Recent researches revealed that cyclophosphamide impacts spermatogenesis by direct DNA and RNA damage and promotes death, as well as affecting Sertoli cell protein expression (Drumond et al., 2011; Pavin et al., 2018; Sadeghzadeh et al., 2020). Our sperm analysis agreed with the 2020 World Health Organization recommendation since we observed no developed sperms in the CYP group (World Health Organization, 2010). All of these causes can promote male infertility *via* impairing sperm function, which is a concern for cancer patients receiving CYP as chemotherapy.

Transplantation of MSCs can enhance the microcirculation of gonadal organ transplantation, boost cell colonisation, and repair the injured somatic parts (Vermeulen et al., 2019). Since bone marrow cells were injected into patients enduring high-dose chemotherapy over 2 decades ago, MSCs have been employed in clinical studies. MSCs produce growth factors and cytokines to promote cell division, inhibit apoptosis, and eventually boost the healing process (Ayatollahi et al., 2014). MSCs heal damaged tissues by direct differentiation-driven cell-for-cell substitution (Pittenger et al., 2019). MSCs can also promote healing by providing mitochondria for the affected cells. Transferring mitochondria is a key mechanism in the deterrence of apoptosis and the reversion of metabolic damage in target cells. MSCs improve antioxidant defence and a global metabolic switch to conserve energy supply on afflicted host cells. In this scenario, MSCs appear to be promising prospects for reducing the negative effects of anti-cancer medications (Gomez-Salazar et al., 2020). Stem cells might help to restore spermatogenesis in two ways: sustaining pre-existing spermatogonial stem cells (SSCs) or transdifferentiating into SSC-like cells to create spermatocytes (Diao et al., 2022).

**FIGURE 7:**
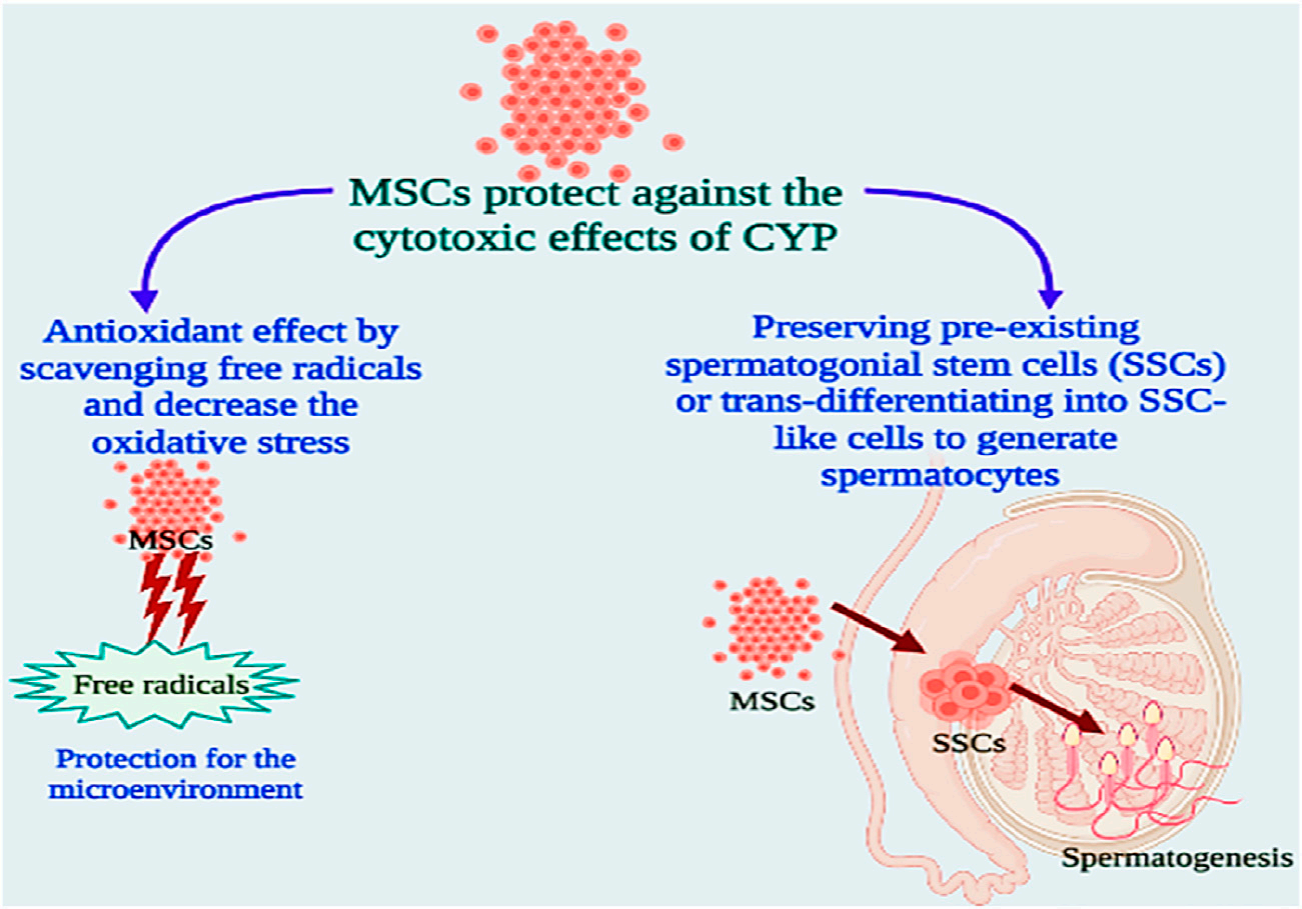
Possible mechanisms for the protective effects of MSCs against cytotoxicity of CYP.

Based on these findings, we tested the alleviative effects of MSCs on CYP-induced infertility in rats by injecting MSCs into the rats’ testes after the last cyclophosphamide dosage. When compared to the CYP group, serum and testicular sex hormones (FSH, LH, and free TH) concentrations were dramatically reduced. These finding suggested that MSCs had a negative affect on the pituitary gland and led to a highly significant reduction in serum FSH and LH in the treated group when compared to the CYP group, and as regeneration of testicular tissue occurred from stem cells, we detected a highly significant rise in the tissue expression of FSH and LH in the treated group when compared with the cyclophosphamide CYP group. Furthermore, when compared with the CYP group, treatment with MSCs following CYP-induced infertile rats resulted in a substantial increase in testicular total antioxidant capacity and testicular SOD enzyme activity. MSCs boosted the antioxidant defence system by scavenging free radicals and decreasing oxidative stress generated by CYP, according to these findings. MSCs have antioxidant capabilities that contribute to their therapeutic effectiveness in a variety of diseases (Figure 7).

The task of MSCs in reducing oxidative and nitrosative damage has lately attracted a lot of attention. MSCs have the capacity to instantly attenuate oxidative stress-related damage, which is associated with cellular damage, inflammation, and disruptive metabolism (Ayatollahi et al., 2014; Gomez-Salazar et al., 2020). MSCs display antioxidant activities either directly (by scavenging free radicals and providing mitochondria) or indirectly (by upregulating antioxidant defences in other cells and changing cellular physiology) (Inan et al., 2017; Stavely and Nurgali, 2020). MSCs can also prevent the production of ROS, hence reducing oxidative stress. MSCs are robust for oxidative and nitrosative stimuli *in vitro*, which has been linked to overexpression of the antioxidant enzymes SOD, catalase, and glutathione peroxidase, along with high amounts of the antioxidant glutathione (Stavely and Nurgali, 2020). It has been suggested that MSCs can prevent oxidative damage by eliminating free radicals, improving host antioxidant defences, moderating the inflammatory reaction, boosting cellular respiration and mitochondrial activities, or offering mitochondria to preserve injured cells (Inan et al., 2017). Additionally, media conditioned by MSCs have high antioxidant capacity, suggesting that MSCs effectively produce antioxidants. Thus, the antioxidant effects of MSCs could be clarified by their capability to direct free radical scavenging as well as by improving antioxidant defences in host tissues *via* antioxidant enzyme upregulation (Stavely and Nurgali, 2020). Recently, MSCs have been considered as one of the greatest sources of stem cell treatment due to their distinct properties, which include rapid proliferation and the ability to specialise into particular cells (Dissanayake et al., 2018; Malekmohamadi et al., 2019; Salem et al., 2019). Sertoli cells and chemicals such as testosterone are employed as inducers of stem cell differentiation into germ-like cells (Zhang et al., 2014; Malekmohamadi et al., 2019). In this work, all testicular cells were employed as an efficient microenvironment to generate germ-like cells isolated from BMSCs. As functional somatic testicular cells, the Sertoli, Leydig, and Myoid cells can impact spermatogenesis and germ cell differentiation. Some metabolic proteins released from Sertoli cells including an insulin-like growth factor and the transformation of growth factors alpha and beta, which are required for germ cell differentiation and spermatogenesis control (Malekmohamadi et al., 2019). According to these results, we examined the testicular tissues histologically following treating CYP-induced infertile rats with MSCs and discovered that MSC transplantation in testes tissues improved the CYP-induced pathological lesions to some degree, with the emergence of a few scattered early spermatids and a few signs of development in most of the seminiferous tubules. After morphometric study for semen analysis, we showed that the spermatogenesis process was slightly improved compared with the CYP-treated group, and the sperms were protected from the toxic effects of CYP. Our results were in accordance with previous studies that proved that stem cells can be developed into germ cells, and spermatogenesis happened after transplanting differentiated germ cells from MSCs into sterile animals testes, and the animals eventually generated spermatozoon primordial germ cells or germ-like cells, which can be created by various cell sources of stem cells such as bone marrow stem cells (Marques-Mari et al., 2009; Vlajković et al., 2012; Ghasemzadeh-Hasankolaei et al., 2016; Salem et al., 2019). All of our data suggest that MSCs can protect against the cytotoxic effects of CYP in producing infertility in male chemotherapy patients that may be by two different mechanisms which may be by preserving pre-existing spermatogonial stem cells (SSCs) or trans-differentiating into SSC-like cells to generate spermatocytes (Kubota and Brinster, 2018; Ibtisham and Honaramooz, 2020; Diao et al., 2022).

## 5 Conclusion

Although CYP is a powerful chemotherapeutic agent, its adverse effects on non-target cells may restrict its usage and raise the probability of CYP cessation. As a result, various researches were carried out in order to discover an appropriate adjunctive medication to offset the negative impacts of CYP. Stem cells have been shown to have powerful antioxidant properties besides germ cell proliferation. The enhancement of oxidative stress indicators provides evidence of MSCs’ protective effect on the antioxidant defence system in cells, and the restoration of MSCs’ harmful effect on testicular tissue revealed that MSCs may be an excellent candidate for treating induced or hereditary infertility. MSCs have a vital role in guarding cell architecture and physiological functioning, as well as reducing CYP-related adverse responses. MSCs have been proven to have a good protective impact and may help to considerably minimise CYP pull-out. In our current research, we observed that CYP administration caused alterations in the biochemical, histological, and microscopic analysis of semen in adult male rats. Our findings show that CYP injection caused testicular tissue damage by raising oxidative stress and influencing sex hormones that control the spermatogenesis process, resulting in infertility in adult male rats. MSC transplantation, on the other hand, resulted in a considerable restoration of sex hormone concentrations by shielding testicular tissues from oxidative damage. Histological and microscopic examination studies corroborated this protective effect, revealing an enhancement in testicular tissue architecture as well as an improvement in the spermatogenesis cycle. Our results indicated that transplanting MSCs had both positive and protective impacts on the spermatogenesis process. As a result, we anticipate that transplanting MSCs to chemotherapy patients will effectively alleviate the spermatogenesis process. As a result, we anticipate that transplanting MSCs to chemotherapy patients will effectively alleviate the spermatogenesis process.

## Data Availability

The original contributions presented in the study are included in the article/Supplementary Material, further inquiries can be directed to the corresponding author.
