# Supplementary material for: Therapeutic potential of bone marrow mesenchymal stem cells in cyclophosphamide-induced infertility
Source: Front Pharmacol. 2023 Mar 22;14:1122175. doi: 10.3389/fphar.2023.1122175 (PMC10073512; doi:10.3389/fphar.2023.1122175)
Supplement: Supplementary file 1 [file Table1.DOCX]

Supporting Information

Therapeutic Potential of Bone Marrow Mesenchymal Stem Cells in Cyclophosphamide-Induced Infertility

Dalia Ibrahim ^1^, Nadia Abozied ^2^, Samar Abdel Maboud ^3^, Ahmad Alzamami ^4^, Norah A Alturki ^5^, Mariusz Jaremko ^6^, Maram Khalil Alanazi ^7^, Hayaa M. Alhuthali ^8^, and Asmaa Seddek ^1^

**Table S1.** Johnsen score for predicting spermatogenesis using cell profile found along the seminiferous

tubules.

| Score | Degree of spermatogenesis |
| --- | --- |
| 1 | No epithelial cells in seminiferous tubules and no tubular sclerosis |
| 2 | No germ cells and presence of Sertoli cells alone |
| 3 | Presence of spermatogonia only |
| 4 | Spermatogenesis is ceased at the initial spermatocyte stage, and just a few spermatogonia are present. |
| 5 | Numerous spermatocytes |
| 6 | Spermatogenesis was interrupted at the spermatid stage due to a lack of early spermatids. |
| 7 | Absence of late spermatids, but there were a lot of early spermatids. |
| 8 | Each tubule contains less than five spermatozoa. |
| 9 | Spermatogenesis is slightly hampered. |
| 10 | Complete spermatogenesis |
